# Pregnancy in Classical Paroxysmal Nocturnal Hemoglobinuria and Aplastic Anemia–Paroxysmal Nocturnal Hemoglobinuria: A High-Risk Constellation

**DOI:** 10.3389/fmed.2020.543372

**Published:** 2020-09-24

**Authors:** Ferras Alashkar, Fuat H. Saner, Colin Vance, Ute Schmücker, Dörte Herich-Terhürne, Ulrich Dührsen, Angela Köninger, Alexander Röth

**Affiliations:** ^1^Department of Hematology, West German Cancer Center, University Hospital Essen, University of Duisburg-Essen, Essen, Germany; ^2^Department of General, Visceral and Transplant Surgery, University Hospital, University Duisburg Essen, Essen, Germany; ^3^Rheinisch-Westfälisches Institut für Wirtschaftsforschung, Essen, Germany; ^4^Department of Gynecology and Obstetrics, University Hospital Essen, University of Duisburg-Essen, Essen, Germany

**Keywords:** eculizumab, pregnancy, thrombosis, aplastic anemia (AA), paroxysmal nocturnal hemoglobinuria (PNH)

## Abstract

Pregnancies in paroxysmal nocturnal hemoglobinuria (PNH) are associated with increased morbidity and mortality. Retrospective studies suggest that outcome has improved with the advent of the complement inhibitor eculizumab. To substantiate this assumption we analyzed the data from patients treated in our department since 2009. All patients were included in the International PNH registry and followed prospectively. We identified 16 pregnancies in 9 patients with classical PNH, and two pregnancies in two patients with aplastic anemia (AA)-PNH. In classical PNH, 13 pregnancies were supported by eculizumab. Breakthrough hemolysis occurred in six pregnancies, necessitating an increase in the biweekly eculizumab dose from 900 mg to 1,200–1,800 mg. Red blood cell transfusions were given in six and platelet transfusions in two pregnancies. A Budd-Chiari syndrome and cholecystitis complicated the course of two pregnancies. Four of 13 pregnancies supported by eculizumab ended in spontaneous abortion or stillbirth, and one was prematurely terminated because of fetal trisomy 21. None of the three pregnancies not supported by eculizumab had a successful outcome. Half the deliveries were preterm. None of the patients died, and, in all but one patient, the post-partum period was uneventful. Both pregnancies in patients with AA-PNH took a favorable course. Our results confirm low maternal mortality and frequent breakthrough hemolysis in pregnant PNH patients receiving eculizumab. Fetal mortality and the rate of preterm delivery were higher than reported previously, possibly related to the use of registry data that are likely to reduce the risk of publication and recall biases.

## Introduction

Paroxysmal nocturnal hemoglobinuria (PNH) is a non-malignant clonal disorder of the pluripotent hematopoietic stem cell with a worldwide incidence of 1–1.5 cases per million individuals (1). It is caused by an acquired mutation of the PIGA gene resulting in reduced or absent expression of the glycosylphosphatidylinositol anchor in all of the stem cell's progeny (1, 2). In erythrocytes, this abnormality affects CD55 and CD59, two membrane-bound inhibitors of the complement system, whose reduced expression sensitizes the cells to chronic complement-mediated intravascular hemolysis with exacerbation at times of increased complement activity, such as infection, trauma, or pregnancy (1). Apart from hemolysis and anemia, PNH is characterized by a strikingly increased risk of thrombosis, often at unusual sites, such as the hepatic, mesenteric, or cerebral veins (3).

In most PNH patients, the stem cell clone carrying the PIGA mutation produces the majority of hematopoietic cells. This situation called classical PNH is characterized by uncontrolled hemolysis and thrombophilia (1, 2). At lower clonal abundance, PNH cells can also be found in bone marrow failure disorders, such as aplastic anemia (AA), and in apparently healthy individuals (1, 2). In the first-named condition referred to as AA-PNH, the disease is dominated by grossly reduced blood cell production rather than hemolysis, and treated according to AA principles (4). The last-named condition is called subclinical PNH (2).

Interestingly, most individuals with subclinical PNH do not progress to overt classical PNH, suggesting that other factors must be involved in disease development (1, 2). Apart from cell-intrinsic clonal evolution, selection pressure exerted by the immune system seems to be of particular importance (1). Most cases of acquired AA are caused by an autoimmune attack on hematopoietic stem cells (4). Cells lacking glycosylphosphatidylinositol-anchored membrane proteins may be less immunogenic, explaining why the proportion of PNH cells may increase in acquired AA. Importantly, cytopenias beyond the erythroid lineage are not only observed in AA-PNH, but also in classical PNH which is often accompanied by reduced blood cell production (1). Evidence has been presented that immune-mediated bone marrow suppression may also be responsible for the expansion of the PNH clone in classical PNH (1). The relationship between PNH and AA-PNH is complex. Not only can AA-PNH develop into classical hemolytic PNH, but, conversely, classical PNH may be followed by AA (1).

Pregnancy is associated with an increased risk of maternal thrombosis (5) and a heightened activity of the complement system (6, 7). Maternal and fetal morbidity and mortality were found to be increased in pregnant PNH patients (3), to an extent to recommend pregnancy avoidance (8). This advice changed with the advent of eculizumab, a monoclonal antibody effectively interrupting the complement cascade and reducing hemolysis and the risk of thrombosis (9). With eculizumab, pregnancy outcomes appear to be more favorable (10, 11). However, due to patient selection and the retrospective nature of the studies so far reported, the effect of eculizumab on pregnancy outcome remains poorly defined.

Here we report pregnancy outcomes in patients with classical PNH or AA-PNH who presented within a 10-year period to the Department of Hematology of the University Hospital Essen in Germany. All consenting patients with a diagnosis of classical PNH or AA-PNH were entered into the International PNH Registry (1) and followed prospectively. In contrast to previous reports (8, 10–12), this is a single-center observational study in which at least part of the data was collected prospectively.

## Materials and Methods

### Study Design and Participants

This is a single-center observational study of pregnancies in women with classical PNH or AA-PNH who were treated at the Department of Hematology of the University Hospital Essen in Germany. After obtaining written informed consent, clinical and laboratory data were documented in the International PNH Registry (1), and the disease course was followed prospectively. Pregnancies occurring before referral to our department were recorded retrospectively based on patient information and medical reports. The study was approved by the Ethics Committee of the University of Duisburg-Essen and conducted in accordance with the Declaration of Helsinki.

### Disease-Related Definitions, Methods, and Treatments

PNH and AA were diagnosed according to international standards. PNH clone size was determined by flow cytometry and expressed as the percentage of fluorescein-labeled proaerolysin-negative granulocytes (2). Treatment indications followed international guidelines (2). After meningococcal vaccination, treatment-requiring PNH patients received 4 weekly infusions of 600 mg eculizumab (induction phase), followed by 900 mg every 2 weeks (maintenance phase). Eculizumab levels were determined by Alexion Pharmaceuticals, Inc, Boston, MA, USA using an enzyme-linked immunosorbent assay that detects both free and complement-bound eculizumab (13).

AA was graded according to established guidelines (14). Patients with severe AA (sAA) received a 4- or 5-day cycle of antithymocyte globulin together with cyclosporine which was maintained for a minimum of 12 months, then tapered according to treatment response (15). Treatment of non-severe AA (nsAA) was restricted to transfusion-dependent patients (14).

### Pregnancy-Related Definitions and Treatments

Spontaneous abortion and stillbirth were defined as unsuccessful pregnancy outcomes before or after gestational week 22, respectively. Preterm delivery was defined as delivery before gestational week 37.

Pregnancy-related breakthrough hemolysis was defined as a drop of the hemoglobin level by 2 g/dL or more with a concomitant increase of the lactate dehydrogenase activity, compared to the latest previous assessment (16). The eculizumab dose was adjusted to maintain a hemoglobin concentration above 8 g/dl throughout pregnancy, sometimes aided by red blood cell transfusions. Platelet transfusions aimed at maintaining a platelet count above 15/nL. All patients received anticoagulation with enoxaparin. The dose was adjusted to platelet count according to personal experience and published recommendations (3). In some cases, anticoagulation was monitored by rotational thromboelastometry (17).

### Statistical Analysis

Due to the small sample size, statistical analysis was not performed.

## Results

Between May 2009 and January 2020, 16 pregnancies in nine patients with classical PNH and two pregnancies in two patients with AA-PNH were recorded at the Department of Hematology of the University Hospital Essen. Although three classical PNH patients had a history of AA, PNH was the leading disease at conception. In patients with AA-PNH, AA was the leading disease. In the following, findings in classical PNH and AA-PNH are presented separately.

### Management of Pregnancy in Classical PNH

PNH developed *de novo* in six and following nsAA or sAA in one and two patients, respectively, with intervals of 96, 23, and 87 months from AA onset. nsAA did not require treatment, and sAA had been treated successfully with standard immunosuppression 23 months or more before conception.

Median age at conception was 29 years (range, 20–34). Six patients had one, two had three, and one had four pregnancies, the fourth being a twin pregnancy (Table 1). The median interval between PNH diagnosis and conception was 45 months. In one patient, PNH was diagnosed 14 months after the first pregnancy, and in another, it was diagnosed in gestational week 11 of the first pregnancy. In the remaining cases, PNH diagnosis preceded pregnancy by 11–119 months, with a median PNH clone size at first pregnancy visit (gestational week 2–17) of 90% (range, 30–100). Except in patient 9, the PNH clone comprised at least two thirds of the hematopoietic system.

**Table 1:** Management of classical paroxysmal nocturnal hemoglobinuria before, during, and after pregnancy.

**Patient (diagnosis)**	**Pregnancy**	**Before pregnancy**		**During pregnancy**				**After pregnancy**
		**Treatment indication**	**Eculizumab dose (mg)**	**Interval PNH diagnosis—conception (months)**	**Major complications**	**Eculizumab dose (mg)^**a**^**	**Blood transfusion**	**Eculizumab dose (mg)**
**Exclusively successful pregnancy outcomes**								
1 (DN)	1st	None	0	−1	Hemolysis	900	8 RBC-U	900
	2nd	Hemolytic anemia	900	21	Breakthrough hemolysis	900–1,800^b^	10 RBC-U	900
	3rd		900	96	Breakthrough hemolysis	1,200–1,800	1 RBC-U	1,200
2 (DN)	1st	Hemolytic anemia	0^c^	63	Breakthrough hemolysis Budd-Chiari syndrome Cholecystitis	900–1,800^b^	37 RBC-U, 2 P-U	900
3 (nsAA)	1st	Hemolytic anemiaBudd-Chiari syndromeSinus vein thrombosis^d^	900	44	Budd-Chiari syndrome Cholecystitis	900	22 RBC-U, 6 P-U	900
4 (DN)	1st	None	0^e^	108	Breakthrough hemolysis	900–1,200	0	900
5 (sAA)	1st	Sinus vein thrombosis	900	66	Breakthrough hemolysis	900–1,200	0	900
**Successful and unsuccessful pregnancy outcomes**								
6 (DN)	1st	Hemolytic anemia	900	11	Spontaneous abortion	0^f^	2 RBC-U	900
	2nd	Hemolytic anemia	900	47	Spontaneous abortion	900	0	900
	3rd	Hemolytic anemia	900	71	Spontaneous abortion	900	0	900
	4th (twins)	Hemolytic anemia	900	119	Breakthrough hemolysis	900–1,200	0	900
**Exclusively unsuccessful pregnancy outcomes**								
7 (DN)	1st	None	0^g^	−14	Spontaneous abortion	0	4 RBC-U	0
	2nd	Portal vein thrombosis	900	40	Stillbirth	900	0	900
	3rd	Portal vein thrombosis	900	51	Medical abortion^h^	900	0	900
8 (DN)	1st	None	0	46	Spontaneous abortion	0	0	0
9 (sAA)	1st	Deep vein thrombosis	900	15	Preeclampsia, stillbirth	900	6 RBC-U	900

PNH, paroxysmal nocturnal hemoglobinuria; DN, de novo PNH; nsAA, PNH after non-severe aplastic anemia; sAA, PNH after severe aplastic anemia; RBC-U, red blood cell unit; P-U, platelet unit.

a Biweekly dose (unless otherwise stated).

b Nine hundred milligrams weekly (instead of 1,800 mg biweekly).

c Glucocorticosteroids and RBC transfusions, no access to eculizumab.

d Four years before diagnosis of PNH, possibly related to oral contraception.

e Splenectomy.

f Eculizumab discontinued because of fear of untoward effects on pregnancy.

g Splenectomy and RBC transfusions for suspected elliptocytosis (incorrect diagnosis).

h Fetal trisomy 21.

Eleven pregnancies were conceived under treatment with eculizumab which had been initiated because of severe hemolysis and/or venous thrombosis (median treatment duration before pregnancy, 50 months; range, 9–117). Patient 2 had no access to eculizumab in her country of origin and had instead been treated with glucocorticosteroids and transfusions. She was the only transfusion-dependent patient in the cohort. Two patients did not require treatment before pregnancy, and in two, the diagnosis of PNH had not been established at the time of first conception (Table 1).

One pregnancy ended in a spontaneous abortion before PNH was diagnosed. In the remaining 15 pregnancies, eculizumab was continued in ten, newly started in the first trimester in three (because of increasing hemolysis), and discontinued in one (for fear of untoward effects). One patient had no indication for eculizumab.

**Figure 1:**
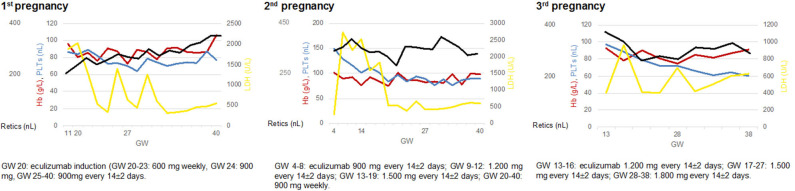
Management of pregnancies complicated by transfusion-dependent breakthrough hemolysis in patient 1. GW, gestational week; Hb, hemoglobin (reference range, 120–152 g/L); LDH, lactate dehydrogenase (120–247 U/L); PLTs, platelets (180–380/nL); Retics, reticulocytes (22–76/nL).

**Figure 2:**
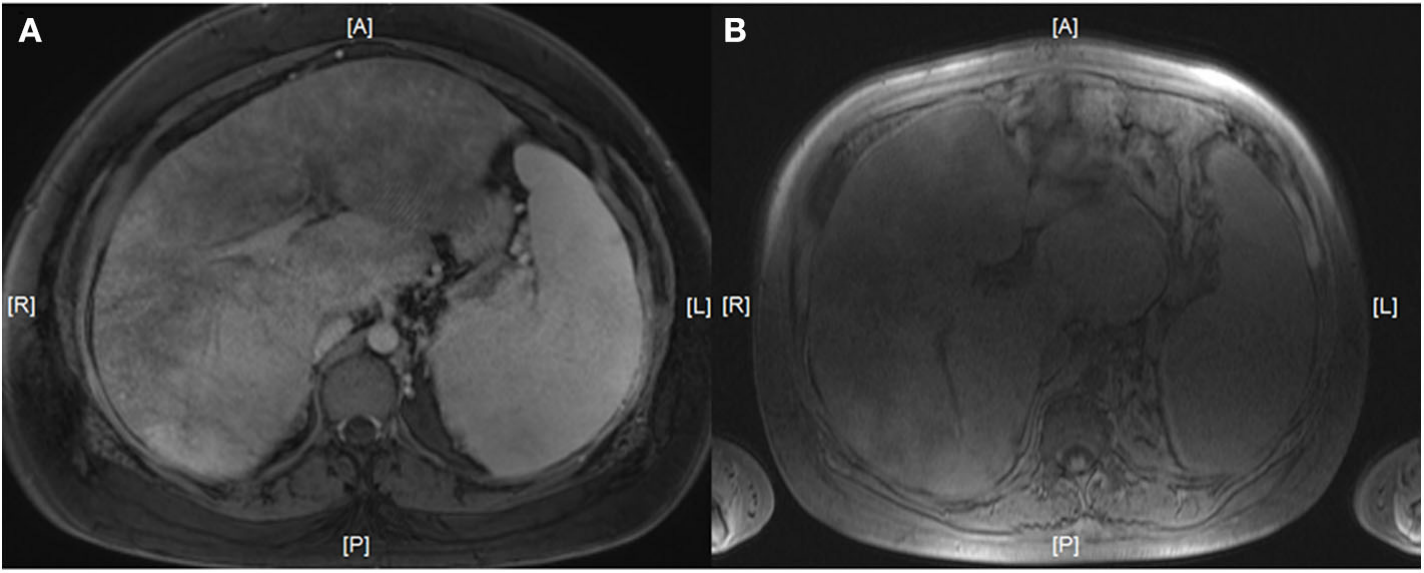
Magnetic resonance imaging showing a (partial) Budd-Chiari syndrome in two PNH patients. Patient 2 (**A**, gestational week 22) and 3 (**B**, gestational week 25). A, anterior; L, left; P, posterior; R, right.

Breakthrough hemolysis occurred in six of 13 pregnancies supported by eculizumab, necessitating a stepwise increase in the biweekly dose to 1,200 mg in three cases and 1,800 mg in another three cases (given either as a single biweekly dose of 1,800 mg or as a weekly dose of 900 mg) (Figure 1). In the remaining cases, a standard dose of 900 mg was continued throughout pregnancy (Table 1). To maintain a hemoglobin level above 8 g/dl, red blood cell transfusions were required in eight pregnancies, one in a patient with an initial misdiagnosis of elliptocytosis, and two in patients with antecedent nsAA or sAA. Two pregnancies were complicated by cholecystitis and a Budd-Chiari syndrome which were successfully managed by antibiotics and anticoagulation, respectively (Figure 2).

To prevent thromboembolic complications, all patients received subcutaneous enoxaparin during and up to 6 months after pregnancy. In general, a prophylactic dose was used, except for patients with a history of thrombosis or Budd-Chiari syndrome (Table 1) who received intermediate-dose or therapeutic anticoagulation. Dosing of enoxaparin was complicated by thrombocytopenia that was seen in all patients with and six patients without antecedent AA (platelet count at first pregnancy visit, 50–177/nL; further decrease to 15–19/nL in two patients with and one patient without antecedent AA; normal range, 180–380/nL). Platelet transfusions were restricted to patients on therapeutic anticoagulation for Budd-Chiari syndrome. Bleeding complications were not observed.

### Maternal, Fetal, and Neonatal Outcomes in Classical PNH

None of the patients died. After pregnancy, eculizumab was gradually tapered to 900 mg every 2 weeks, except for one patient who required 1,200 mg to control hemolysis after her third pregnancy (Table 1). In the post-partum period, both patients with Budd-Chiari syndrome and the patient giving birth to twins received red blood cell transfusions (2, 7, and 2 units, respectively), and both Budd-Chiari patients and a patient undergoing medical abortion for fetal trisomy 21 followed by curettage received platelet transfusions (2, 6, and 1 unit, respectively). Patient 1 developed cholecystitis and choledocholithiasis after the first of her three successful pregnancies which were treated by endoscopic cholecystectomy and papillotomy. Six months after delivery, PNH was well-controlled in all patients, and blood transfusions were no longer required.

Eight of the 16 pregnancies (including a twin pregnancy) had a successful outcome (Table 2), with delivery of five male and four female newborns. Five of these deliveries were preterm due to premature labor, cholecystitis, fetal distress, or premature rupture of membranes (twin pregnancy). Eight pregnancies had an unsuccessful outcome, with spontaneous abortion in five, medical abortion in one, and stillbirth in two cases. In one patient, stillbirth was preceded by preeclampsia (Table 1). The placentas of both patients suffering stillbirth were examined histologically showing thromboses, infarcts, and fibrosis compatible with placental insufficiency. Similar histological abnormalities were found in the placenta of the patient whose first pregnancy antedated the diagnosis of PNH by 14 months and ended in a spontaneous abortion, suggesting that PNH had already been present at the time of abortion. There was no obvious correlation between pregnancy outcome and maternal age, PNH duration, duration of eculizumab therapy, PNH clone size, extent of hemolysis, or thrombotic complications (Table 1). None of the patients experiencing spontaneous abortion or stillbirth was investigated for other causes of pregnancy loss.

**Table 2:** Delivery and status of newborns in women with classical paroxysmal nocturnal hemoglobinuria or aplastic anemia–paroxysmal nocturnal hemoglobinuria.

**Patient**	**Pregnancy**	**Delivery**		**Newborn**			
		**Time^**a**^**	**Type**	**Apgar score^**b**^**	**Weight (g)**	**Height (cm)**	**Head circumference (cm)**
**Classical paroxysmal nocturnal hemoglobinuria**							
1	1st	40 + 3	Vaginal	5/8/10	3,750	54	36
	2nd	40 + 2	Vaginal	8/9/10	3,850	54	37
	3rd	38 + 5	Vaginal	9/10/10	3,870	53	36
2	1st	35 + 3	Cesarean section	9/10/10	2,380	46	32
3	1st	31 + 2	Cesarean section	7/8/9	1,750	44	30.5
4	1st	35 + 7	Cesarean section	6/8/9	3,050	49	34
5	1st	39 + 1	Vaginal	9/10/10	2,745	47	33
6	4th (twins)	32 + 5	Cesarean section	9/9/10	1,780	43	29.5
		32 + 5	Cesarean section	8/9/10	1,900	47	31
**Aplastic anemia–paroxysmal nocturnal hemoglobinuria**							
1	1st	39 + 1	Vaginal	9/10/10	3,020	50	35
2	2nd	35 + 2	Cesarean section	9/10/10	2,520	45	33

a Gestational week plus days.

b *After 1, 5, and 10 min*.

Excluding the pregnancy that occurred 14 months before PNH was diagnosed and the pregnancy that was terminated for a medical reason, eight of 14 pregnancies (57%) had a successful and six (43%) had an unsuccessful outcome. Limiting the analysis to pregnancies supported by eculizumab, eight of 13 (62%) were successful and five (38%) were not.

Eculizumab levels were determined in one patient and her newborn. With a biweekly dose of 1,500 mg, the mother's serum contained eculizumab levels exceeding 100 μg/ml, but the antibody was not detected in breast milk or cord blood (<35 μg/ml).

### Management and Outcomes of Pregnancies in AA-PNH

In the first AA patient, pregnancy started 53 months after sAA was diagnosed and successfully treated by standard immunosuppression. The patient had a small PNH clone of 9.5%. At her first pregnancy visit, the blood values were normal except for slight thrombocytopenia (125/nl) which remained constant during the subsequent course. There were no signs of hemolysis. To control AA a low dose of cyclosporine (50 mg daily) was continued throughout pregnancy. Both pregnancy and delivery of a healthy male newborn were uneventful (Table 2).

The second patient was diagnosed with nsAA in gestational week 29 of her second pregnancy. The first pregnancy's outcome had been successful, but details were not recorded. The patient had an almost acellular bone marrow, severe anemia (hemoglobin 5.5 g/dl) and thrombocytopenia (12/nl), and a PNH clone of 18% with signs of hemolysis. Because of increasing transfusion dependency, immunosuppression with cyclosporine was initiated in gestational week 32. Delivery was preterm, with birth of a healthy male newborn (Table 2). Because of persistent pancytopenia the patient received standard immunosuppression with antithymocyte globulin and cyclosporine in the post-partum period, resulting in a complete remission.

## Discussion

Both PNH and AA are conditions with a negative impact on pregnancy. The results of our single-center study confirm some, but not all of the observations made by other investigators.

With regard to pregnancy in PNH, numerous case histories have been reported, but, to our knowledge, there are only four retrospective studies that include higher patient numbers. Two studies, comprising 43 and 25 pregnancies, respectively, summarized the findings in the pre-eculizumab era (8, 12), while the other two, comprising 75 and 14 pregnancies, focused on treatment of pregnant patients with eculizumab (10, 11). The most important finding confirmed by our study is that, with the advent of eculizumab and more widespread use of anticoagulation, maternal mortality has decreased from 8–12% to 0%. This is associated with a decrease of potentially lethal post-partum thrombosis from 14–16% to 0–3%. A salutary effect of eculizumab on other PNH-related complications is less evident. During pregnancy, the proportion of patients requiring red blood cell (36–58% vs. 36–50%) or platelet transfusions (21–36% vs. 7–21%) and the proportion of patients whose pregnancy is complicated by thrombosis (0–7% vs. 0–13%), hemorrhage (4–7% vs. 0–3%), or (pre)eclampsia (9% vs. 8–14%) appears similar in the pre- and post-eculizumab eras. The same is true for post-partum hemorrhage (4–9% vs. 0–14%). The rate of breakthrough hemolysis in our study (46%) did not differ from that previously reported in pregnant patients receiving eculizumab (36–48%). Vaginal delivery (as opposed to cesarean section) was chosen in 55–70% of patients in the pre-eculizumab era and in 36–51% in the post-eculizumab era (all data taken from the literature (8, 10–12) and the present study).

Our study differs from previous reports in a higher fetal and neonatal mortality and a higher rate of preterm delivery. Among 15 pregnancies not terminated for a medical reason, we observed five spontaneous abortions (33%) two of which occurred in patients receiving eculizumab (2/12, 17%). By contrast, spontaneous abortions were not reported in the studies summarizing pregnancy outcomes in the pre-eculizumab era (8, 12), and they amounted to 0 and 8% in the two publications dealing with the post-eculizumab era (10, 11). The rate of stillbirth also appeared to be increased in our cohort (13%) compared to the publications covering the pre- and post-eculizumab eras (4–7% and 0–4%, respectively). Preterm deliveries were observed in 56% of successful pregnancies in our study, as compared to 29–39% and 27–29% in the previous publications (8, 10–12).

Apart from small numbers and the possibility that some patients may have had unidentified medical conditions favoring pregnancy loss unrelated to PNH, patient selection is the most likely reason for the observed discrepancies. Two of the previous publications were compilations of published case reports (8, 11), and the other two were based on questionnaires sent out to hematologists (10, 12). Publication and recall biases are likely to have influenced patient selection (18). The present study was based on documentation of our patients' findings in the International PNH Registry. While only part of the data was collected prospectively, data loss due to publication or recall bias was less likely to occur than in the retrospective studies published before.

Pregnancies in patients with AA-PNH appeared to be associated with fewer complications than pregnancies in classical PNH. Patient numbers, however, were small, and the spectrum of AA was restricted to patients with a PNH clone. With the introduction of immunosuppression, pregnancy outcomes have improved significantly in AA (19, 20). In a retrospective study, more than half of pregnancies took an uneventful course. Premature birth was observed in 14%, and maternal and fetal death was rare (20). Our study confirms these findings. It also shows that AA may arise or worsen in the course of pregnancy (14, 20).

To our knowledge, this is the largest single-center experience of pregnancies in PNH so far reported. Our data confirms previous evidence that maternal mortality is low in patients receiving eculizumab. Fetal mortality and preterm delivery, however, may be higher than previously reported. Prospective studies are warranted to confirm our findings.

## Data Availability Statement

All datasets generated for this study are included in the article/supplementary material.

## Ethics Statement

The studies involving human participants were reviewed and approved by retrospective analysis and use of data was approved by the Ethical Committee of the Faculty of Medicine at the University Hospital of Duisburg-Essen and the study was conducted in accordance to the Declaration of Helsinki and written patients' informed consent was obtained from all patients. The patients/participants provided their written informed consent to participate in this study.

## Author Contributions

FA and AR conceived the study. FA, FHS, US, DH-T, UD, AK, and AR directed the clinical activities. FA, CV, and AR directed the research activities. FA and UD wrote the manuscript. All authors interpreted the data and gave final approval of the manuscript.

## Conflict of Interest

UD and AR received honoraria from Alexion Pharmaceuticals and Roche Pharma. The remaining authors declare that the research was conducted in the absence of any commercial or financial relationships that could be construed as a potential conflict of interest.
